# Cass Co. (Ind.) Medical Society

**Published:** 1875-08

**Authors:** I. B. Washburne

**Affiliations:** Sec.


Adjourned.

---

THE CASS COUNTY (IND.) MEDICAL SOCIETY.

The Society met in the room of Hall's Business College, in Logansport, June 30, 1875. The President, Dr. A. Coleman, in the chair.

After the usual routine business, the clinic was opened by Dr. W. H. Bell, presenting a case of softening of the tibia, which was prescribed for.

Dr. Adrain introduced a case of ulcerated cornea, which was examined and discussed briefly by several of the members.

Another case was presented by Dr. Adrain, who asked the society to examine and give him the etiology, pathology and therapeutics of it.

Dr. Fitch said it belonged to a class of cases which he recognized as an "Anomalous disease of the Tongue," in a letter to the *North-Western Medical Journal*, in 1838. It was epidemic then, and cases had been seen occasionally since that time.

Dr. Coleman said he had treated the case before the Society, and he recognized it as belonging to the class of cases spoken of by Dr. Fitch, and the same that he had described in a short paper read before the Society the past year. It commences with a stinging or burning pain in the tongue, the tip and edges are red and may become fissured, the saliva is hot and scalding, the papilla and epithelium slip off, and the tongue is left smooth. The patient may be able to work, and possess a good appetite, but only the mildest food can be taken. It may change to the soles of the feet, with like symptoms there. The treatment is not satisfactory, though tonics had done good, and creasote, he thought, beneficial. The patients improved slowly, but generally recovered in the summer season.

Dr. Adrain thought it belonged to the dartrous diathesis, that a skin disease generally preceded it, and that alteratives would act well.

Dr. Adrain then read a paper on cerebro-spinal meningitis, which was discussed by Drs. Bell, Fitch, Coleman, Adrain, and Faber.

The third annual election of officers was then held, and resulted in the selection of—

Dr. Graham N. Fitch, President.

R. Faber, Vice-President.

I. B. Washburne, Secretary.

J. M. Justice, Treasurer.

J. A. Adrain, Wm. H. Bell, and J. Z. Powell, Censors.

The Society then adjourned to meet Wednesday, Oct. 6th, 1875.

I. B. Washburne, M.D., Sec.

